# Treatment of Ascarides

**Published:** 1868-08

**Authors:** 


### Treatment of Ascarides.

Three correspondents write to the *Lancet* as follows, in reply to an inquiry as to the best treatment for ascarides. (Quoted by the *Medical Gazette*, New York.)

1.—First, empty the intestine as thoroughly as possible by injecting with warm soap-and-water, then (supposing the patient to be a child of five or six years of age) throw into the bowel two drachms of tincture of iron dissolved in two or three ounces of very strong infusion of quassia, and repeat it at intervals of two days if necessary. The state of the general health must be improved, and the vitiated mucus secretion of the intestine, in which the worms burrow, be dislodged by a few grains of calomel at bedtime, followed by a dose of salts in the morning. Afterwards tonics, as the syrup of the phosphate of quinia, strychnia, and iron will most likely be required.

2.—Tincture of perchloride of iron (B. P., 1867,) five drachms; infusion of quassia, fifteen and a half ounces; mix: two tablespoonfuls to be taken three times a day. Two drachms of tincture of perchloride of iron, to be used as an injection in half a pint of thin starch every third night.

3.—The most prompt and effectual is the best olive oil, one tablespoonful at bedtime, with jalap powder and scammony in the morning; doses according to age. I have treated many cases in this way, and have never known it to fail. The dose given twice a week will be quite sufficient.

